# The Involvement of Nek2 and Notch in the Proliferation of Rat Adrenal Cortex Triggered by POMC-Derived Peptides

**DOI:** 10.1371/journal.pone.0108657

**Published:** 2014-10-03

**Authors:** Pedro Omori Ribeiro de Mendonca, Ismael Cabral Costa, Claudimara Ferini Pacicco Lotfi

**Affiliations:** Department of Anatomy, Institute of Biomedical Sciences, University of São Paulo, São Paulo, SP, Brazil; University of Turin, Italy

## Abstract

The adrenal gland is a dynamic organ that undergoes constant cell turnover. This allows for rapid organ remodeling in response to the physiological demands of the HPA axis, which is controlled by proopiomelanocortin (POMC)-derived peptides, such as adrenocorticotropic hormone (ACTH) and N-Terminal peptides (N-POMC). In the rat adrenal cortex, POMC-derived peptides trigger a mitogenic effect, and this process increases cyclins D and E, while inhibiting p27Kip1. The goal of the present study was to further explore the mitogenic effect of ACTH and synthetic N-POMC_1–28_ peptides by investigating the differences in the expression of key genes involved in the cell cycle of the rat adrenal cortex, following inhibition of the HPA axis. Moreover, we evaluated the differences between the inner and outer fractions of the adrenal cortex (ZF-fraction and ZG-fraction) in terms of their response patterns to different stimuli. In the current study, the inhibition of the HPA axis repressed the expression of *Ccnb2*, *Camk2a*, and *Nek2* genes throughout the adrenal cortex, while treatments with POMC-derived peptides stimulated Nek2, gene and protein expression, and Notch2 gene expression. Furthermore, Notch1 protein expression was restricted to the subcapsular region of the cortex, an area of the adrenal cortex that is well-known for proliferation. We also showed that different regions of the adrenal cortex respond to HPA-axis inhibition and to induction with POMC-derived peptides at different times. These results suggest that cells in the ZG and ZF fractions could be at different phases of the cell cycle. Our results contribute to the understanding of the mechanisms involved in cell cycle regulation in adrenocortical cells triggered by N-POMC peptides and ACTH, and highlight the involvement of genes such as *Nek2* and *Notch*.

## Introduction

The adrenal gland is composed of two distinct regions: the cortex and the medulla. The cortex consists of three concentric zones: the zona glomerulosa (ZG), the zona fasciculata (ZF), and the zona reticularis (ZR). The adrenal cortex requires stimuli from the adrenocorticotropic hormone (ACTH) precursor pro-opiomelanocortin (POMC) for adrenocortical maintenance and remodeling. Several other peptides are secreted from the anterior pituitary, including pro-gamma-MSH. Pro-gamma-MSH potentiates the steroidogenic actions of ACTH, triggering an increase of cholesterol and cholesterol ester hydroxylase activity [1]–[3]. Moreover, the N-terminal sequences of pro-gamma-MSH that do not contain the MSH-gamma sequence (known as the N-terminal portion of POMC, or N-POMC) are able to induce cell proliferation in the adrenal cortex [4] [5]. Previous studies have shown that N-POMC_1–28_ and N-POMC_2–54_ peptides are potent mitogens, both *in* *vitro* [4] and *in* *vivo* [6]. Recent work from our group has shown that synthetic N-POMC_1–28_ induced *in* *vivo* S-phase entry in the entire rat adrenal cortex by up-regulating cyclins D and E [7] [8]. Moreover, N-POMC_1–28_ induced proliferation of rat adrenal cells in primary culture via the ERK1/2 pathway [9].

It is well-known that inhibition of the HPA axis by dexamethasone (DEX) or hypophysectomy can cause atrophy only in the innermost portion of the adrenal cortex [10]–[13]. However, the mechanisms involved in the regulation of cell cycle genes in adrenal cells after the absence of POMC-derived peptides remain poorly understood. This study aimed to evaluate the expression pattern of 86 genes associated with cell cycle regulation in the adrenal cortex of dexamethasone-treated rats after administration of ACTH or synthetic N-POMC_1–28_ peptides (these N-POMC peptides contain correctly aligned disulfide bonds in cysteines [N-POMC^Cys^] or a linear structure with methionines [N-POMC^Met^]). Moreover, we evaluated the differences between the inner and outer fractions of the adrenal cortex in terms of their response patterns to different stimuli, as well as the differences and similarities between the proliferative effects triggered in the adrenal cortex by ACTH and N-POMC peptides. Our results highlight the involvement of some key genes in adrenocortical cellular proliferation, such as *Nek2* and *Notch*.

## Materials and Methods

### Animals

Male Sprague-Dawley rats with an average weight of 250±30 g were obtained from the Institute of Biomedical Sciences of the University of São Paulo and maintained in a temperature-controlled environment and 12-h light/dark cycle. The study was approved by the Animal Experimentation Ethics Committee at the University’s Institute of Biomedical Sciences. The animals were fed with standard rat food and received water *ad libitum*. All experimental procedures were conducted between 9 and 11 am; the rats were euthanized by decapitation and adrenal glands were harvested.

### N-POMC_1–28_ peptide synthesis

N-POMC_1–28_ peptides were synthesized with cysteine (N-POMC^Cys^) or methionine (N-POMC^Met^). The N-POMC^Cys^ peptide was synthesized and characterized by Bachem America Inc. (Torrance, CA, USA) and contained correctly aligned disulfide bridges in the cysteine 2–24 and 8–20 positions. In order to avoid undefined disulfide bonds, the N-POMC^Met^ peptide was synthesized and characterized in the Department of Biochemistry and Biophysics at the Federal University of São Paulo (UNIFESP) by replacing cysteine with methionine.

### Dexamethasone, ACTH, and N-POMC_1–28_ treatments

The animals were treated as previously described in [8]. Briefly, a concentration of 50 µg/100 g of body weight (BW) of Dexamethasone (DEX) (Aché Laboratórios Farmacêuticos, Campinas, SP, Brazil) was administered intraperitoneally, once a day at 9 am for 2 days, with the purpose of inhibiting the HPA axis. The control animals received saline injections and followed the same treatment regimen as the experimental animals. Subsequently, DEX-treated rats were divided into four groups (n = 4–5) and received a single injection of ACTH, N-POMC^Cys^, N-POMC^Met^, or saline. The ACTH, N-POMC^Cys^, and N-POMC^Met^ treatments were administered at a concentration of 10^−7^ M in the dose of 100 µl/100 g BW.

### Preparation of protein lysates

The preparation of protein lysates was performed as described in [7]. Briefly, at 6 hours post treatment, the adrenal glands were removed and gently decapsulated to separate the capsule/zona glomerulosa (ZG fraction, composed mainly of ZG cells) from the zona fasciculata/reticularis and medulla (ZF fraction, composed mainly of ZF cells). The medulla was removed from the ZF fraction and the samples were lysed in ice-cold RIPA and protease inhibitors. Protein concentrations were quantified with the Bradford assay. Previously published data concerning the quality of preparation and separation of the adrenal fractions, showed that the contamination between different fractions was minimal and acceptable [7].

### Nek2 protein expression

Total lysate protein samples from the ZG and ZF fractions (30 µg from each) were sorted in 10% SDS-PAGE. To test the Nek2 protein isoforms, we used a commercial HeLa nuclear extract (Santa Cruz Biotechnology, Santa Cruz, CA, USA) for positive control of the antibody. We also obtained Y1 and primary rat adrenal cell culture total protein extracts, as described in [9]. After electrophoresis, the gel was electroblotted onto Hybond-C nitrocellulose membranes using a semi-dry Bio-Rad apparatus. Non-specific sites were blocked overnight at 4°C with 5% dried non-fat milk dissolved in TBS (150 mM NaCl, 10 mM Tris, 1% Tween 20, pH 7.5). The membranes were subsequently incubated with anti-Nek2 (1∶1000; Santa Cruz) or β-actin (1∶2000, Santa Cruz) for two hours at room temperature. Proteins were detected using chemiluminescent secondary peroxidase-conjugated anti-rabbit or anti-mouse polyclonal antibodies (ECL-Amersham-Pharmacia, Piscataway, NJ, USA). The immunoblot results were quantified by densitometry using the Gel-Pro Imager and Gel-Pro Imager kit Version 1.0 quantification program for Windows. The average and standard error were calculated for the data obtained from each fraction. Ponceau staining was used to monitor protein transfer amounts and total protein loaded.

### Immunohistochemistry of Nek2 and Notch expression

The rats were euthanized 6 h after the final treatments; adrenal glands were removed, fixed in 4% paraformaldehyde in a solution of 0.1 M phosphate buffered saline (PBS; pH 7.4) for 8 h at room temperature, and subsequently immersed in 6% sucrose PBS for 12 h. The fixed and deparaffinized sections were rehydrated through a graded ethanol series, washed with sterile Milli-Q water, PBS, and PBS with 0.5% hydrogen peroxide. Sections were incubated for 10 minutes in boiling citrate buffer (pH 6.0) for antigen retrieval, and the background staining was blocked using a commercial blocker solution (Background Blocker; Diagnostic BioSystems, Pleasanton, CA, USA). These sections were washed and incubated in PBS with anti-Nek2 1∶200 (Santa Cruz), or anti-Notch 1 1∶100 (Cell Signaling Technology, Inc. Danvers, MA, USA) or anti-Notch 2 1∶100 (Cell Signaling) or even Notch 3 1∶500 (Cell Signaling). The immune complexes were detected by immunoperoxidase staining using the Vectastain Elite ABC kit (Vector Laboratories, Burlingame, CA, USA) and visualized with diaminobenzidine (Sigma Fast; Sigma Aldrich, St. Louis, MO, USA) followed by counterstaining with Harris’ hematoxylin and a saturated solution of lithium carbonate. The adrenal sections incubated in non-immune primary sera tested negative (data not shown).

### Total RNA isolation and PCR array

Two hours after final treatments with Saline, ACTH, or N-POMC peptides, the rats were decapitated and their adrenal glands were removed and separated into ZG and ZF fractions. The total RNA was isolated using Trizol extraction according to the manufacturer’s instructions (Life Technologies, USA). The cDNA synthesis was prepared according to the PCR array manufacturer’s instructions (Sabiosciences – Qiagen, Germantown, MD USA). cDNA was mixed with SYBER Green MIX provided by the manufacturer and applied in the PCR array plates (Rat Cell Cycle PARN-020). The assays were performed on a standard real-time PCR device ABI7500 (Applied Biosystems – Life Technology). Data were analyzed using online software provided by the PCR array manufacturer.

### Statistical data analyses

For the Western-Blotting assay, statistical comparisons of three independent immunoblot results were tested using analysis of variance (ANOVA) followed by the Tukey-Kramer Multiple Comparisons Test. For the PCR array experiments, the p values were calculated based on a Student’s t-test of the replicate 2∧(−Delta Ct) values for each gene in the control and treatment groups. Values of *p*≤0.05 were considered statistically significant. The results corresponded to three independent experiments.

### Ethics Statement

This study was carried out in full accordance with the ethical principles of animal experimentation adopted by the Brazilian Society of Laboratory Animals in Science and was approved by the ethics committee on animal use of the Institute of Biomedical Sciences under protocol number 156, page 112, book 02.

## Results

### HPA-axis inhibition reduced *Nek2*, *Ccnb2*, and *Camk2b* expression in the entire cortex

**Figure 1:**
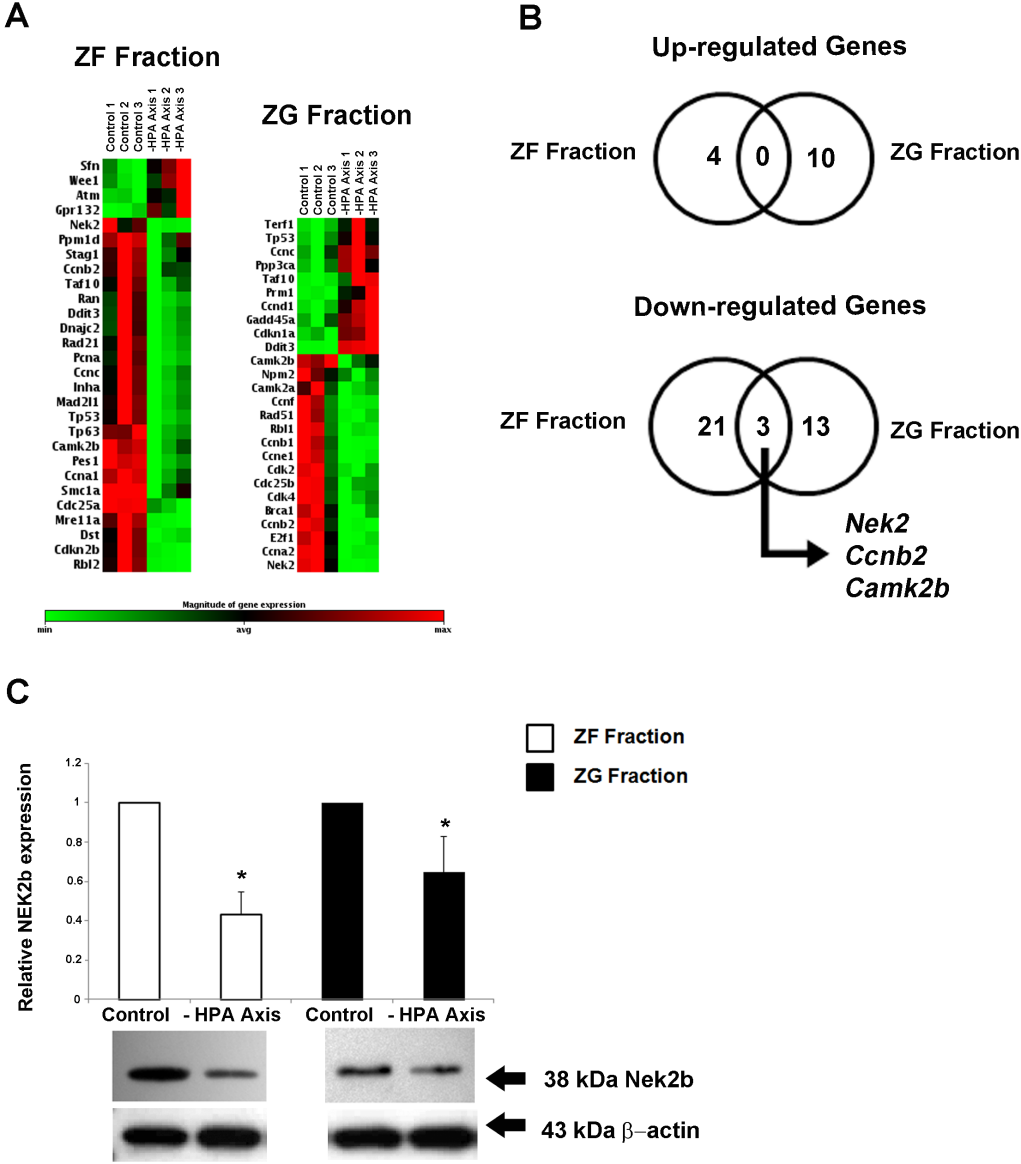
Clustergram showing the expression profile of significantly (p<0.05) altered genes related to the cell cycle in adrenal ZF fraction or ZG fraction after inhibition of the HPA axis with DEX for two days (50 µg/100 g BW) (A) Correlation of up- or down-regulated genes in ZF fraction and ZG fraction; genes regulated in both DEX and control treatments are presented in the figure intersection (B). Analysis of Nek2b protein expression in adrenal ZF fraction and ZG fraction after inhibition of HPA axis with DEX (C). Adrenal glands were separated in two fractions (ZG and ZF), and total protein or total RNA, respectively, was extracted for the immunoblotting or PCR array analysis. Control animals received saline only (Control). *p<0.05, n = 3.

**Figure 2:**
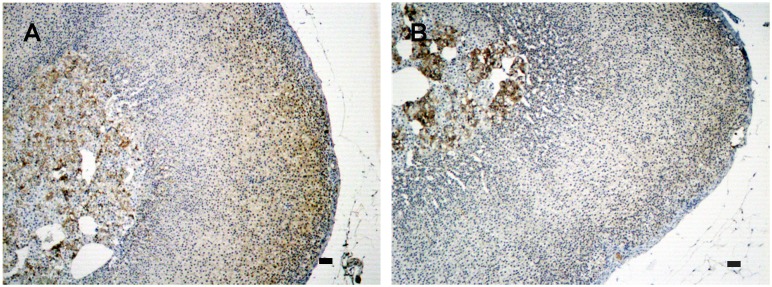
Immunolocalization of Nek2b expression in the adrenal cortex after HPA inhibition. Adrenal sections were obtained from rats treated with: (A) saline only or (B) DEX for two days (50 µg/100 g BW). Nek2b-positive cells were stained brown. Sections were counterstained with Harris’ hematoxylin and differentiated with a saturated solution of lithium carbonate. Bars 10 µm.

To evaluate the effects of HPA-axis inhibition by dexamethasone (DEX) in the expression of genes involved in the cell cycle of the adrenal cortex, we used a real-time RT-PCR-based plate assay. The ZG and ZF fractions showed distinct profiles of gene expression after HPA-axis inhibition with Dex treatment (Figure 1A). Among all significantly regulated genes, three genes were significantly (p<0.05) down-regulated in both fractions of the cortex: *Nek2*, *Ccnb2*, and *Camk2b* (Figure 1B). *Nek2* was the most repressed gene, irrespective of the adrenal fraction (ZG: −17.8 fold and ZF: −12.4 fold). All significantly regulated genes are listed in Table 1. In order to validate the expression of *Nek2* on a protein level, we first evaluated the protein isoform found in the rat adrenal cortex. Our results show that the isoform 2 (Nek2b ∼38 kDa) was expressed in the rat adrenal gland (Figure S1). The expression of Nek2b was reduced in both fractions after HPA-axis inhibition: 0.57-fold and 0.36-fold in the ZG and ZF fractions, respectively (Figure 1C). Immunohistochemistry revealed no expression of Nek2b in the capsule, and a decreased expression in the entire cortex following HPA-axis inhibition (Figure 2).

**Table 1:** Genes significantly regulated in the ZG and ZF fractions of the adrenal cortex after inhibition of the HPA axis.

ZG Fraction After HPA Axis Inhibition			
Gene Symbol	Gene	Fold Regulation	p-value
*Tp53*	Tumor protein p53	1.9386	0.034216
*Cdkn1a*	Cyclin-dependentkinase inhibitor 1A	1.879	0.001773
*Ccnd1*	Cyclin D1	1.7471	0.005476
*Ccnc*	Cyclin C	1.7451	0.009108
*Terf1*	Telomeric repeatbinding factor(NIMA-interacting) 1	1.6837	0.040843
*Ddit3*	DNA-damageinducible transcript 3	1.6415	0.000001
*Taf10*	TAF10 RNA polymeraseII. TATA box bindingprotein (TBP)-associated factor	1.5	0.033599
*Gadd45a*	Growth arrest andDNA-damage-inducible. alpha	1.4692	0.005312
*Prm1*	Protamine 1	1.454	0.022124
*Ppp3ca*	Protein phosphatase 3.catalytic subunit. alpha isoform	1.4439	0.018036
*Camk2a*	Calcium/calmodulin-dependentprotein kinase II alpha	−1.6702	0.041303
*Npm2*	Nucleophosmin/nucleoplasmin 2	−1.7715	0.031786
*Camk2b*	Calcium/calmodulin-dependentprotein kinase II beta	−1.9611	0.012154
*Cdk2*	Cyclin dependent kinase 2	−2.1535	0.046664
*Rbl1*	Retinoblastoma-like 1 (p107)	−2.5462	0.044093
*Cdk4*	Cyclin-dependent kinase 4	−2.5491	0.046248
*Cdc25b*	Cell division cycle 25homolog B (*S. pombe*)	−2.7069	0.041175
*Ccnf*	Cyclin F	−3.5677	0.046238
*Brca1*	Breast cancer 1	−3.5925	0.014395
*Ccne1*	Cyclin E1	−3.6723	0.033051
*Rad51*	RAD51 homolog(RecA homolog.*E. coli*) (*S. cerevisiae*)	−4.302	0.048942
*E2f1*	E2F transcription factor 1	−5.6962	0.0074
*Ccna2*	Cyclin A2	−7.2518	0.01107
*Ccnb1*	Cyclin B1	−9.2642	0.025219
*Ccnb2*	Cyclin B2	−12.8468	0.014697
*Nek2*	NIMA (never in mitosis gene a)-related expressed kinase 2	−17.8973	0.006768
**ZF Fraction After HPA Axis Inhibition**			
**Gene Symbol**	**Gene**	**Fold Regulation**	**p-value**
*Wee1*	Wee 1 homolog (*S. pombe*)	3.5431	0.030867
*Gpr132*	G protein-coupled receptor 132	3.1711	0.023048
*Atm*	Ataxia telangiectasiamutated homolog (human)	2.2763	0.031384
*Sfn*	Stratifin	2.0922	0.015251
*Smc1a*	Structural maintenanceof chromosomes 1A	−1.2058	0.00984
*Stag1*	Stromal antigen 1	−1.2354	0.037677
*Ppm1d*	Protein phosphatase 1Dmagnesium-dependent.delta isoform	−1.3755	0.047927
*Pes1*	escadillo homolog 1.containing BRCT domain (zebrafish)	−1.429	0.000259
*Cdc25a*	Cell division cycle 25homolog A (*S. pombe*)	−1.434	0.000137
*Dst*	Dystonin	−1.5637	0.008329
*Rad21*	RAD21 homolog (*S. pombe*)	−1.6472	0.015744
*Ccnc*	Cyclin C	−1.6644	0.021064
*Rbl2*	Retinoblastoma-like 2	−1.7112	0.004351
*Mre11a*	MRE11 meiotic recombination11 homolog A (*S. cerevisiae*)	−1.8404	0.001225
*Ddit3*	DNA-damage inducible transcript 3	−1.8661	0.046409
*Camk2b*	Calcium/calmodulin-dependentprotein kinase II beta	−1.8725	0.002969
*Ccna1*	Cyclin A1	−1.8856	0.00258
*Dnajc2*	DnaJ (Hsp40) homolog.subfamily C. member 2	−1.9862	0.047149
*Mad2l1*	MAD2 mitotic arrestdeficient-like 1 (yeast)	−2.0209	0.011379
*Taf10*	TAF10 RNA polymerase II.TATA box binding protein(TBP)-associated factor	−2.0491	0.032413
*Ran*	RAN. member RAS oncogene family	−2.166	0.032983
*Tp53*	Tumor protein p53	−2.1735	0.010038
*Ccnb2*	Cyclin B2	−2.3784	0.032474
*Cdkn2b*	Cyclin-dependent kinaseinhibitor 2B (p15. inhibits CDK4)	−2.7226	0.005969
*Tp63*	Tumor protein p63	−2.7702	0.0034
*Inha*	Inhibin alpha	−2.8879	0.016507
*Pcna*	Proliferating cell nuclear antigen	−2.9079	0.019438
*Nek2*	NIMA (never in mitosis gene a)-relatedexpressed kinase 2	−12.4235	0.01

### ACTH and N-POMC peptides induce distinct gene expression profiles in the outer and inner zones of the adrenal cortex

**Figure 3:**
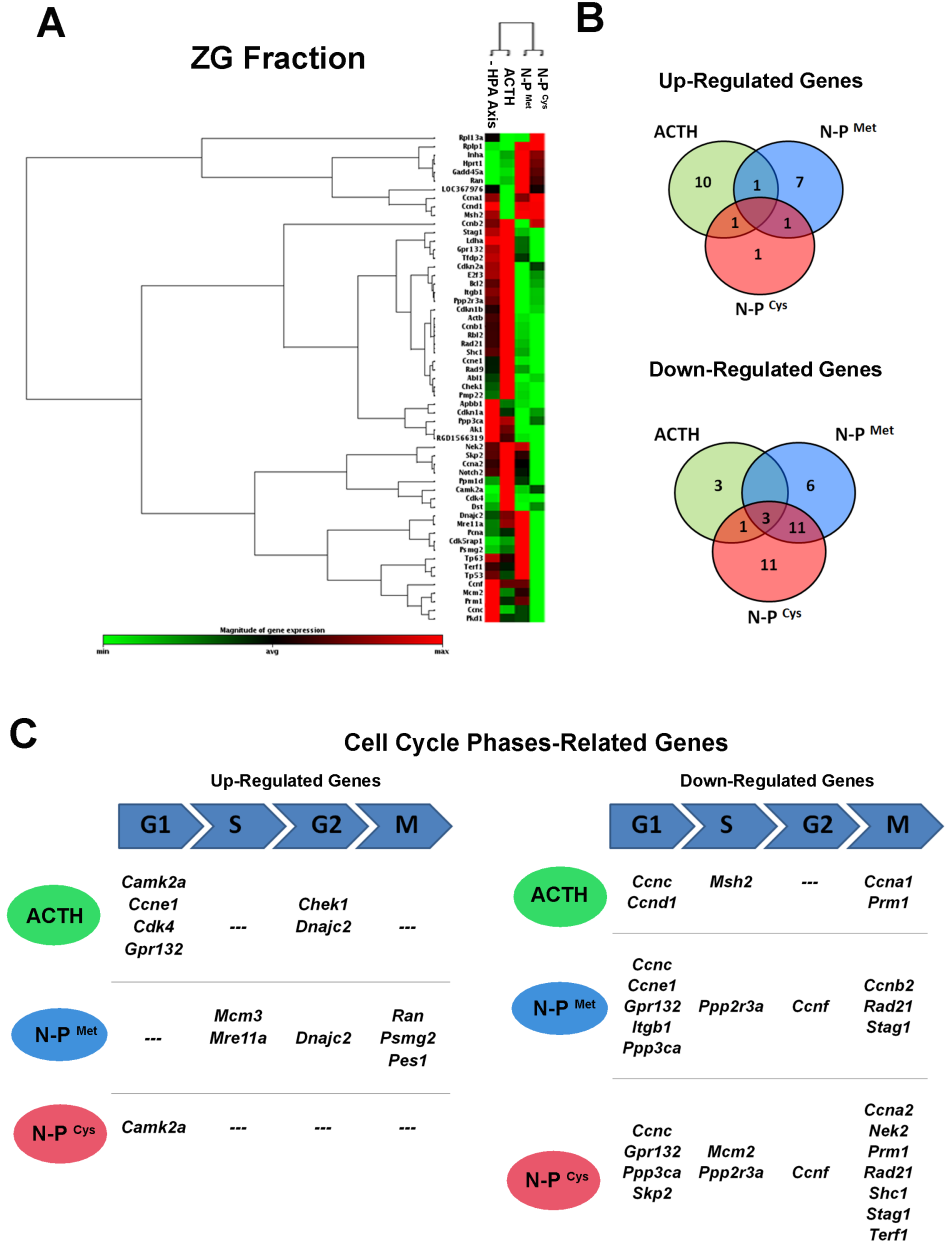
Results of the PCR array for cell cycle related genes in the ZG fraction. Clustergram showing the expression profile of significantly (p<0.05) altered genes related to the cell cycle in the adrenal ZG fraction after inhibition of HPA axis with DEX for two days (50 µg/100 g BW), followed by ACTH or N-POMC^Met^ or N-POMC^Cys^ treatments (A). Correlation of up- or down-regulated genes in the ZG fraction after treatments, showing the number of genes regulated by both treatments with POMC-derived peptides (B). Up- or down-regulated genes related to specific cell cycle phases after ACTH, N-POMC^Met^, or N-POMC^Cys^ treatments (C). G1: G1 phase and G1/S transition; G2: G2 phase; S: S phase and DNA replication; M: M phase.

**Figure 4:**
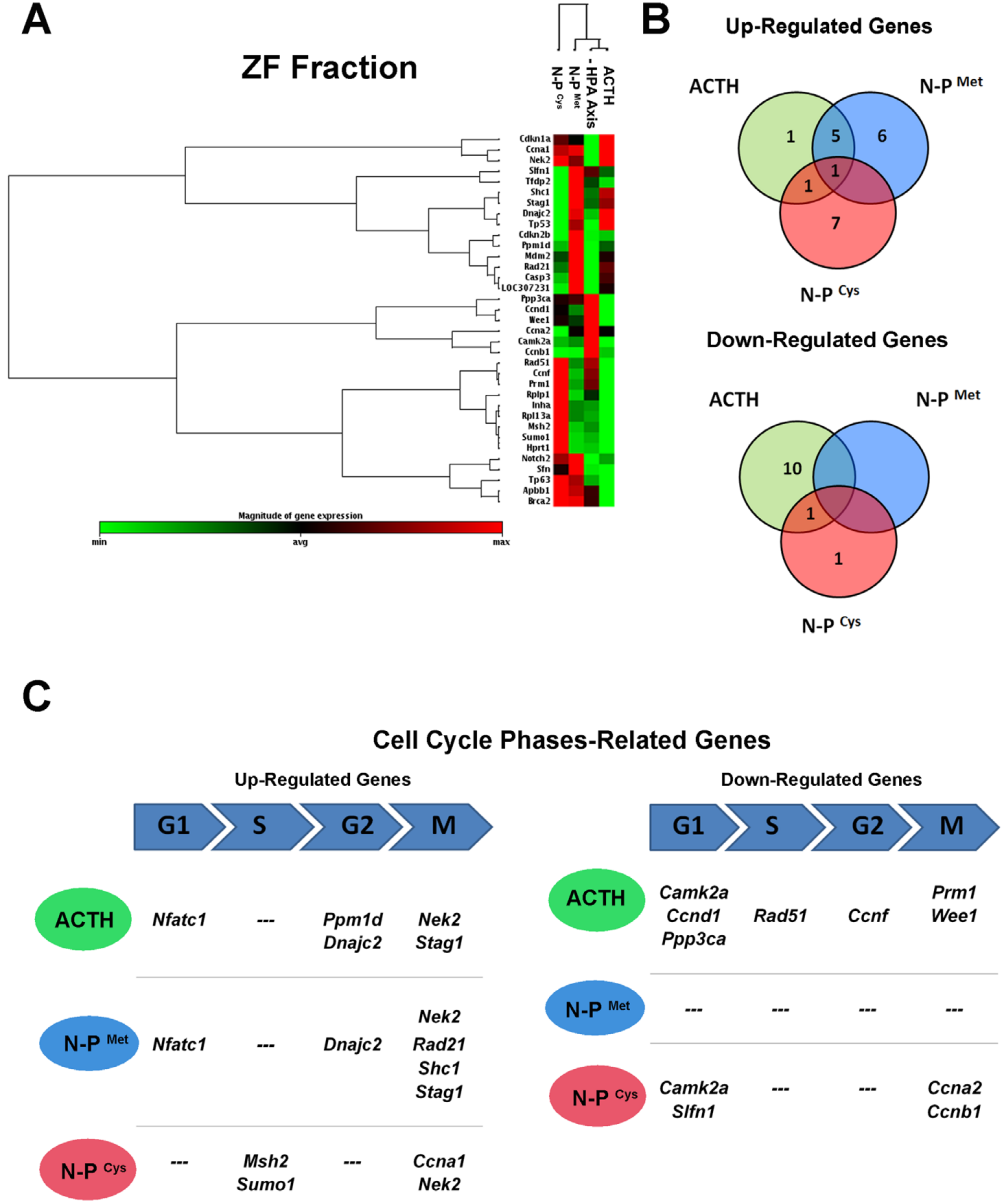
Results of the PCR array for cell cycle related genes in the ZF fraction. Clustergram showing the expression profile of significantly (p<0.05) altered genes related to the cell cycle in the adrenal ZF fraction after inhibition of the HPA axis with DEX for two days (50 µg/100 g BW), followed by ACTH, N-POMC^Met^, or N-POMC^Cys^ treatments (A). Correlation of up- or down-regulated genes in the ZF fraction after treatments, showing the number of genes regulated by both treatments with POMC-derived peptides (B). Up- or down-regulated genes related to specific cell cycle phases after ACTH, N-POMC^Met^, or N-POMC^Cys^ treatments (C). G1: G1 phase and G1/S transition; G2: G2 phase; S: S phase and DNA replication; M: M phase.

We report results for treatments with ACTH and N-POMC peptides on genes that were significantly (p<0.05) up- or down-regulated. In the ZG fraction, treatment with ACTH promoted a distinct profile of gene expression compared to treatment with N-POMC peptides (Figure 3A). These distinct responses could be partially due to the larger number of repressed genes in common between treatments with the two N-POMC peptides (14) compared to those repressed in common between treatment with ACTH and either N-POMC peptide (4). Only three genes were down-regulated in all treatments: *Ccnc*, *Cdkn1a*, and *Pkd1*. Our data also reveal that ACTH up-regulated the expression of *Camk2* (as did N-POMC^Cys^) and of *Dnajc2* (as did N-POMC^Met^). Moreover, *Inha* expression was up-regulated after stimulation with both N-POMC peptides (Figure 3B). When we analyzed cell cycle-related genes, we observed that ACTH up-regulated four genes related to the G1 phase (*Camk2a*, *Ccne1*, *Cdk4*, and *Gpr132*) and two related to the G2 phase (*Chek1* and *Dnajc2*). Treatment with N-POMC^Met^ up-regulated the expression of genes related to the S phase (*Mcm3* and *Mre11a*), the G2 phase (*Dnajc2*), and the M phase (*Ran*, *Psmg2*, and *Pes1*), whereas N-POMC^Cys^ up-regulated the expression of only one gene (*Camk2a*), which is related to the G1 phase (Figure 3C). Furthermore, N-POMC^Cys^ down-regulated more genes (7) related to a late phase of the cell cycle (M-phase) than ACTH (2) or N-POMC^Met^ (3). The list of all significantly altered genes in the ZG fraction, including their fold regulation data, is available in Table 2. In the ZF fraction, the clustergram based only on significantly altered genes reveals that N-POMC^Cys^ treatment promoted a distinct pattern of gene expression when compared to the N-POMC^Met^ and ACTH treatments (Figure 4A). Also, when we analyzed the genes that were regulated in common by the different treatments, we observed that *Nek2* was up-regulated in all three treatments. *Stag1, Mdm2, Casp3, Dnajc2*, and *Nfatc1* were up-regulated after treatment with both ACTH and N-POMC^Met^, whereas *Ppm1d* was up-regulated after treatment with either ACTH or N-POMC^Cys^. Among the genes down-regulated in common between the treatments with POMC-derived peptides, only *Camk2a* was repressed after treatment with ACTH or N-POMC^Cys^. Interestingly, N-POMC^Met^ did not repress the expression of any gene (Figure 4B). When we analyzed genes related to specific phases of the cell cycle, we observed that ACTH up-regulated genes related to the G1 phase (*Nfatc1*), the G2 phase (*Ppm1d* and *Dnajc2*), and the M phase (*Nek2* and *Stag1*). N-POMC^Met^ up-regulated genes related to the G1 phase (*Nfatc1*), the G2 phase (*Dnajc2*), and the M phase (*Nek2*, *Rad21*, *Shc1*, and *Stag1*). On the other hand, N-POMC^Cys^ treatment up-regulated the expression of genes related to the S phase (*Msh2* and *Sumo1*) and the M phase (*Ccna1* and *Nek2)*. Concomitantly with these up-regulations, ACTH treatment repressed genes related to the G1 phase (*Camk2a*, *Ccnd1*, and *Ppp3ca*), the S phase (*Rad51*), the G2 phase (*Ccnf*), and the M phase (*Prm1* and *Wee1*). Also, N-POMC^Cys^ treatment down-regulated genes related to the G1 phase (*Camk2a* and *Slfn1*) and the M phase (*Ccna2* and *Ccnb1*), as shown in Figure 4C. All significantly altered genes in the ZF fraction, including their fold regulation data, are listed in Table 3.

**Table 2:** Genes significantly regulated in the ZG fraction of the adrenal cortex after inhibition of the HPA axis followed by treatments with ACTH, N-POMC^Met^, or N-POMC^Cys^.

ZG Fraction: ACTH			
Gene Symbol	Gene	Fold Regulation	p-value
*Camk2a*	Calcium/calmodulin-dependent protein kinase II alpha	3.0417	0.000054
*Cdk4*	Cyclin-dependent kinase 4	1.9117	0.011247
*Dst*	Dystonin	1.8402	0.013247
*Ppm1d*	Protein phosphatase 1D magnesium-dependent. delta isoform	1.8084	0.006493
*Pmp22*	Peripheral myelin protein 22	1.58	0.004287
*Rad9*	RAD9 homolog (*S. pombe*)	1.5261	0.026327
*Chek1*	CHK1 checkpoint homolog (*S. pombe*)	1.3946	0.038051
*Ccne1*	Cyclin E1	1.3866	0.044238
*Abl1*	C-abl oncogene 1. receptor tyrosine kinase	1.3409	0.010445
*Dnajc2*	DnaJ (Hsp40) homolog. subfamily C. member 2	1.2555	0.035768
*Notch2*	Notch homolog 2 (Drosophila)	1.1974	0.010077
*Gpr132*	G protein-coupled receptor 132	1.0942	0.024599
*Ccnc*	Cyclin C	−1.4141	0.042834
*Cdkn1a*	Cyclin-dependent kinase inhibitor 1A	−1.4708	0.024637
*Ccnd1*	Cyclin D1	−1.5074	0.02811
*Prm1*	Protamine 1	−1.5085	0.038924
*Msh2*	MutS homolog 2 (*E. coli*)	−1.6135	0.027143
*Pkd1*	Polycystic kidney disease 1 homolog (human)	−1.8617	0.000053
*Ccna1*	Cyclin A1	−2.5344	0.012597
			
**ZG Fraction: N-POMC^Met^**			
**Gene Symbol**	**Gene**	**Fold Regulation**	**p-value**
*Mre11a*	MRE11 meiotic recombination 11 homolog A (*S. cerevisiae*)	1.8348	0.021743
*Psmg2*	Proteasome (prosome. macropain) assembly chaperone 2	1.824	0.007304
*Pcna*	Proliferating cell nuclear antigen	1.6431	0.000977
*Pes1*	Pescadillo homolog 1. containing BRCT domain (zebrafish)	1.598	0.047391
*Inha*	Inhibin alpha	1.5662	0.016083
*Dnajc2*	DnaJ (Hsp40) homolog. subfamily C. member 2	1.4394	0.037783
*Cdk5rap1*	CDK5 regulatory subunit associated protein 1	1.4382	0.003531
*LOC367976*	Similar to DNA replication licensing factor MCM3(DNA polymerase alphaholoenzyme-associated protein P1) (P1-MCM3)	1.3475	0.021984
*Ran*	RAN. member RAS oncogene family	1.2062	0.020662
*Ccnb2*	Cyclin B2	−1.2335	0.012467
*Ccnc*	Cyclin C	−1.2537	0.014855
*Ccnf*	Cyclin F	−1.2646	0.031889
*Ccne1*	Cyclin E1	−1.4087	0.034054
*Bcl2*	B-cell CLL/lymphoma 2	−1.4235	0.034361
*Rad21*	RAD21 homolog (*S. pombe*)	−1.4703	0.001876
*Rbl2*	Retinoblastoma-like 2	−1.4703	0.017071
*Gpr132*	G protein-coupled receptor 132	−1.4714	0.000172
*Stag1*	Stromal antigen 1	−1.4994	0.018796
*Ppp3ca*	Protein phosphatase 3. catalytic subunit. alpha isoform	−1.5327	0.007328
*Cdkn1b*	Cyclin-dependent kinase inhibitor 1B	−1.5434	0.020993
*Ppp2r3a*	Protein phosphatase 2. regulatory subunit B”. alpha	−1.5667	0.010109
*Itgb1*	Integrin. beta 1	−1.7364	0.009488
*Ak1*	Adenylate kinase 1	−1.8606	0.000275
*E2f3*	E2F transcription factor 3	−1.8675	0.011247
*RGD1566319*	Similar to Sestrin 2 (Hi95)	−1.8804	0.000178
*Apbb1*	Amyloid beta (A4) precursor protein-binding. family B. member 1 (Fe65)	−1.8871	0.015075
*Pkd1*	Polycystic kidney disease 1 homolog (human)	−1.9341	0.00016
*Cdkn1a*	Cyclin-dependent kinase inhibitor 1A	−2.0881	0.007196
*Cdkn2a*	Cyclin-dependent kinase inhibitor 2A	−2.202	0.010674
			
**ZG Fraction: N-POMC^Cys^**			
**Gene Symbol**	**Gene**	**Fold Regulation**	**p-value**
*Camk2a*	Calcium/calmodulin-dependent protein kinase II alpha	1.7687	0.001738
*Gadd45a*	Growth arrest and DNA-damage-inducible. alpha	1.594	0.031285
*Inha*	Inhibin alpha	1.455	0.014103
*Ppp3ca*	Protein phosphatase 3. catalytic subunit. alpha isoform	−1.311	0.028009
*Ppp2r3a*	Protein phosphatase 2. regulatory subunit B”. alpha	−1.4132	0.022207
*Terf1*	Telomeric repeat binding factor (NIMA-interacting) 1	−1.433	0.023569
*Ccnc*	Cyclin C	−1.4546	0.016437
*Tfdp2*	Transcription factor Dp-2 (E2F dimerization partner 2)	−1.4614	0.017254
*E2f3*	E2F transcription factor 3	−1.5025	0.016275
*Shc1*	SHC (Src homology 2 domain containing) transforming protein 1	−1.5025	0.01852
*Itgb1*	Integrin. beta 1	−1.5447	0.023496
*Rbl2*	Retinoblastoma-like 2	−1.5735	0.032006
*Notch2*	Notch homolog 2 (Drosophila)	−1.5881	0.002803
*Ccna2*	Cyclin A2	−1.6122	0.012739
*Stag1*	Stromal antigen 1	−1.6178	0.028532
*Rad21*	RAD21 homolog (*S. pombe*)	−1.6594	0.014072
*Mcm2*	Minichromosome maintenance complex component 2	−1.6613	0.013286
*Cdkn1a*	Cyclin-dependent kinase inhibitor 1A	−1.7021	0.028311
*Rad9*	RAD9 homolog (*S. pombe*)	−1.7319	0.04898
*Skp2*	S-phase kinase-associated protein 2 (p45)	−1.7621	0.029069
*Ak1*	Adenylate kinase 1	−1.8412	0.000319
*RGD1566319*	Similar to Sestrin 2 (Hi95)	−1.9417	0.000214
*Apbb1*	Amyloid beta (A4) precursor protein-binding. family B. member 1 (Fe65)	−1.9757	0.01365
*Gpr132*	G protein-coupled receptor 132	−1.9779	0.000082
*Tp53*	Tumor protein p53	−2.1997	0.003929
*Prm1*	Protamine 1	−2.2694	0.007761
*Ccnf*	Cyclin F	−3.3807	0.000503
*Nek2*	NIMA (never in mitosis gene a)-related expressed kinase 2	−3.8655	0.002508
*Pkd1*	Polycystic kidney disease 1 homolog (human)	−4.1911	0.000004
*Tp63*	Tumor protein p63	−4.8422	0.000526

**Table 3:** Genes significantly regulated in the ZF fraction of the adrenal cortex after inhibition of the HPA axis followed by treatments with ACTH, N-POMC^Met^, or N-POMC^Cys^.

ZF Fraction: ACTH			
Gene Symbol	Gene	Fold Regulation	p-value
*Nek2*	NIMA (never in mitosis gene a)-related expressed kinase 2	3.3299	0.015864
*LOC307231*	Similar to nuclear factor of activated T-cells. cytoplasmic. calcineurin-dependent 1	2.6109	0.027357
*Ppm1d*	Protein phosphatase 1D magnesium-dependent. delta isoform	1.5109	0.000879
*Casp3*	Caspase 3	1.3435	0.022633
*Tp53*	Tumor protein p53	1.3065	0.001567
*Stag1*	Stromal antigen 1	1.1732	0.045706
*Dnajc2*	DnaJ (Hsp40) homolog. subfamily C. member 2	1.1606	0.003126
*Mdm2*	Mdm2 p53 binding protein homolog (mouse)	1.0758	0.009491
*Inha*	Inhibin alpha	−1.2838	0.046751
*Tp63*	Tumor protein p63	−1.4785	0.006269
*Msh2*	MutS homolog 2 (*E. coli*)	−1.5469	0.000832
*Rad51*	RAD51 homolog (RecA homolog. *E. coli*) (*S. cerevisiae*)	−1.5965	0.040077
*Ppp3ca*	Protein phosphatase 3. catalytic subunit. alpha isoform	−1.704	0.04028
*Ccnd1*	Cyclin D1	−1.7342	0.040145
*Wee1*	Wee 1 homolog (*S. pombe*)	−1.8408	0.014263
*Camk2a*	Calcium/calmodulin-dependent protein kinase II alpha	−2.2419	0.016674
*Ccnf*	Cyclin F	−2.6841	0.04902
*Apbb1*	Amyloid beta (A4) precursor protein-binding. family B. member 1 (Fe65)	−2.6949	0.0375
*Prm1*	Protamine 1	−3.3064	0.049452
**ZF Fraction: N-POMC^Met^**			
**Gene Symbol**	**Gene**	**Fold Regulation**	**p-value**
*LOC307231*	Similar to nuclear factor of activated T-cells. cytoplasmic. calcineurin-dependent 1	3.9279	0.00014
*Cdkn2b*	Cyclin-dependent kinase inhibitor 2B	3.2928	0.045492
*Notch2*	Notch homolog 2 (Drosophila)	2.8467	0.043297
*Nek2*	NIMA (never in mitosis gene a)-related expressed kinase 2	2.7511	0.001391
*Casp3*	Caspase 3	1.5415	0.038784
*Shc1*	SHC (Src homology 2 domain containing) transforming protein 1	1.2958	0.042992
*Rad21*	RAD21 homolog (*S. pombe*)	1.257	0.023741
*Tp53*	Tumor protein p53	1.2521	0.002109
*Stag1*	Stromal antigen 1	1.248	0.021117
*Dnajc2*	DnaJ (Hsp40) homolog. subfamily C. member 2	1.152	0.001929
*Mdm2*	Mdm2 p53 binding protein homolog (mouse)	1.1368	0.030173
*Brca2*	Breast cancer 2	1.1087	0.040819
**ZF Fraction: N-POMC^Cys^**			
**Gene Symbol**	**Gene**	**Fold Regulation**	**p-value**
*Nek2*	NIMA (never in mitosis gene a)-related expressed kinase 2	3.3058	0.011916
*Tp63*	Tumor protein p63	2.7895	0.008098
*Ccna1*	Cyclin A1	2.4794	0.007717
*Msh2*	MutS homolog 2 (*E. coli*)	2.3214	0.018572
*Sumo1*	SMT3 suppressor of mif two 3 homolog 1 (*S. cerevisiae*)	2.2038	0.025152
*Inha*	Inhibin alpha	1.9185	0.000318
*Sfn*	Stratifin	1.8277	0.000008
*Cdkn1a*	Cyclin-dependent kinase inhibitor 1A	1.2397	0.016317
*Ppm1d*	Protein phosphatase 1D magnesium-dependent. delta isoform	1.21	0.024761
*Ccnb1*	Cyclin B1	−1.4845	0.039232
*Camk2a*	Calcium/calmodulin-dependent protein kinase II alpha	−1.9252	0.024671
*Ccna2*	Cyclin A2	−2.1361	0.026184
*Slfn1*	Schlafen 1	−2.2423	0.043104

### ACTH and N-POMC^Met^ increase Nek2b expression in the entire adrenal cortex

**Figure 5:**
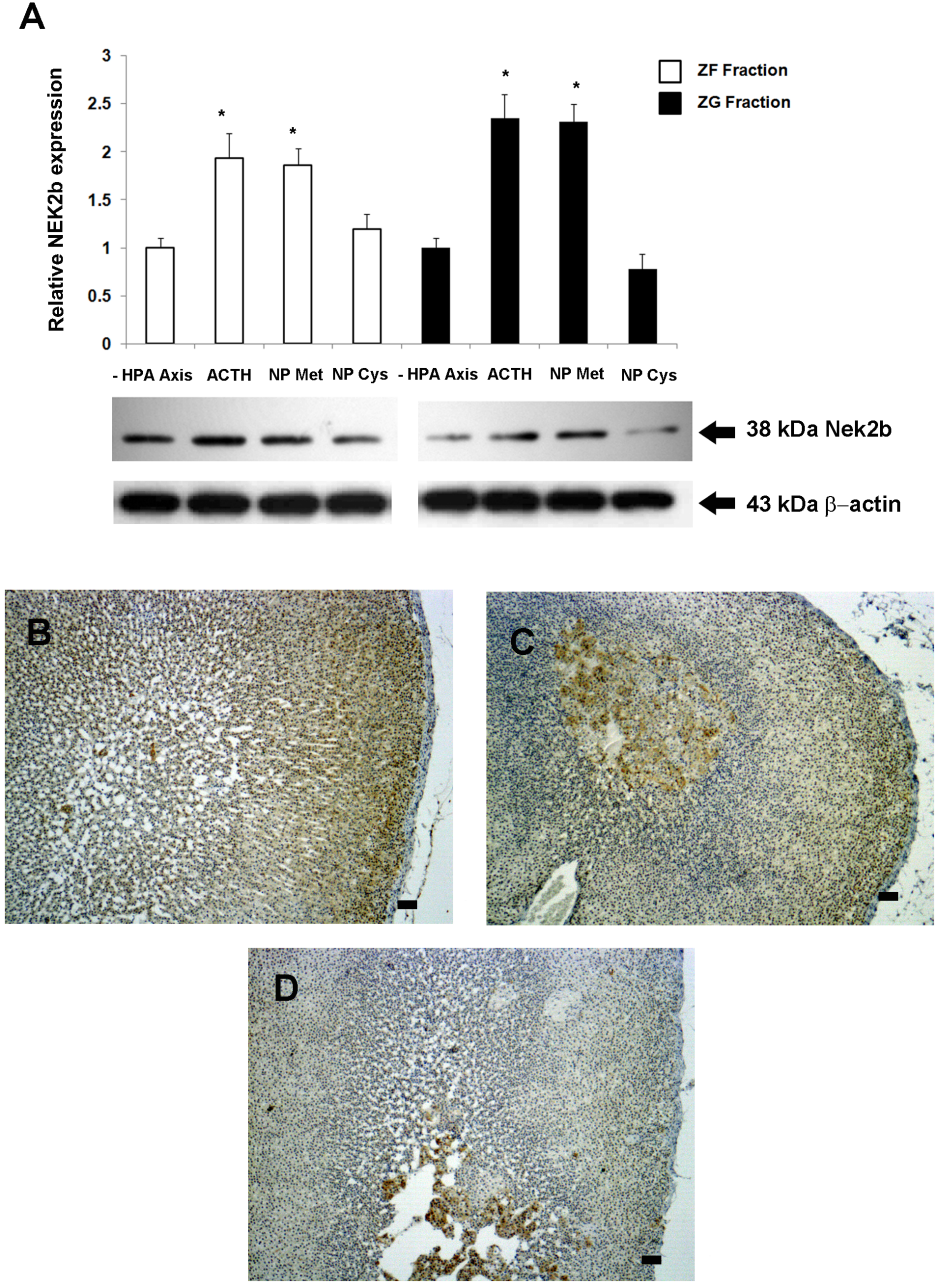
Analysis of Nek2b protein expression in adrenal ZF fraction and ZG fraction after inhibition of the HPA axis with DEX, followed by ACTH, N-POMC^Met^, or N-POMC^Cys^ treatments. Adrenal glands were separated in two fractions (ZG and ZF), and total protein was extracted for immunoblotting analysis (A). Immunolocalization of Nek2b expression in the adrenal cortex after HPA inhibition followed by ACTH (B), N-POMC^Met^ (C), or N-POMC^Cys^ (D) treatments. Adrenal sections were obtained from rats treated previously with DEX for two days (50 µg/100 g BW). Nek2b-positive cells were stained brown. Sections were counterstained with Harris’ hematoxylin and differentiated with a saturated solution of lithium carbonate. Bars 10 µm. *p<0.05, n = 3.

Based on previous data with PCR array, we chose Nek2 to evaluate the variation in the expression of some candidate genes on a protein level. This gene appeared down-regulated in the entire adrenal cortex after inhibition of the HPA axis and all three treatments up-regulated its expression in the ZF fraction. After treatments with either ACTH or N-POMC^Met^, there was an increase in Nek2b expression in the ZG fraction (2.35-fold and 2.31-fold, respectively) and also in the ZF fraction (1.93-fold and 1.85-fold, respectively) (Figure 5A). Immunohistochemistry showed increased Nek2b expression in the entire cortex following treatments with ACTH and N-POMC^Met^ (Figure 5B and C). On the other hand, N-POMC^Cys^ treatment did not alter Nek2b expression, irrespective of the adrenal fraction analyzed (Figure 5D).

### The expression of Notch proteins in the adrenal gland

**Figure 6:**
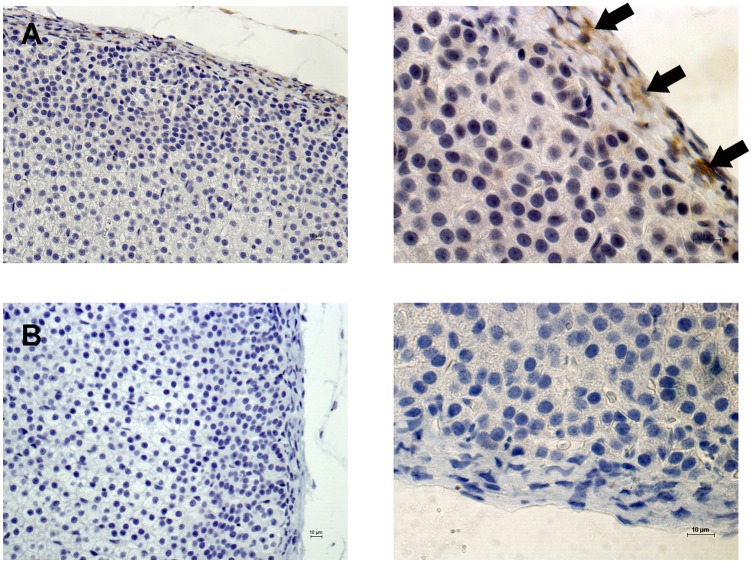
Immunolocalization of Notch2 expression in the adrenal cortex. Adrenal sections were obtained from rats treated with saline only. Notch2-positive cells are stained brown and indicated by arrows (A). Negative control without primary antibody (B). Sections were counterstained with Harris’ hematoxylin and differentiated with a saturated solution of lithium carbonate. Bars 10 µm.

**Figure 7:**
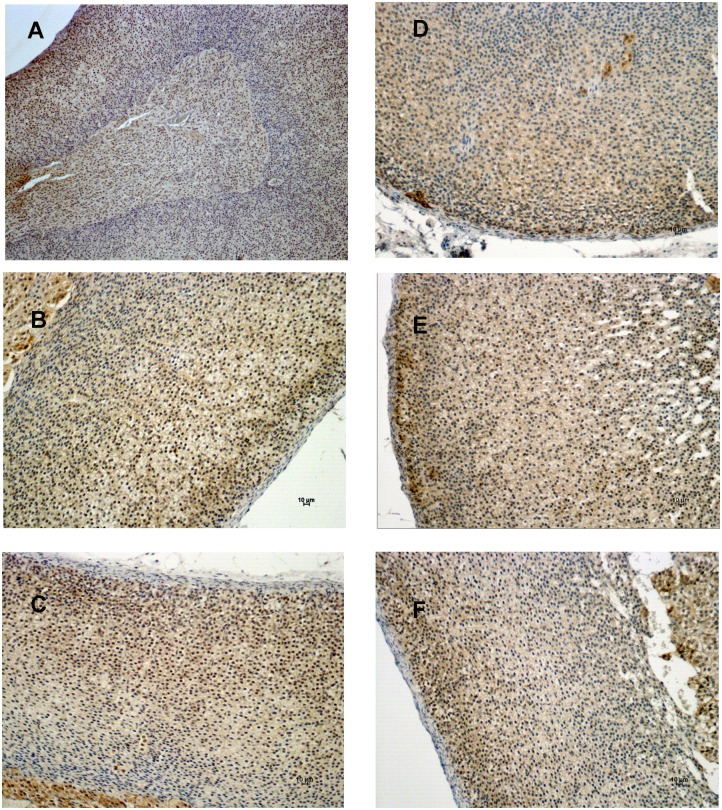
Immunolocalization of Notch1 expression in the adrenal cortex. Adrenal sections were obtained from rats treated with saline only (A and B) or two days with DEX (50 µg/100 g BW) followed by: saline (C), or ACTH (D), or N-POMC^Met^ (E), or even N-POMC^Cys^ (F). Notch1-positive cells are stained in brown and indicated by arrows. Sections were counterstained with Harris’ hematoxylin and differentiated with a saturated solution of lithium carbonate. Bars 10 µm.

**Figure 8:**
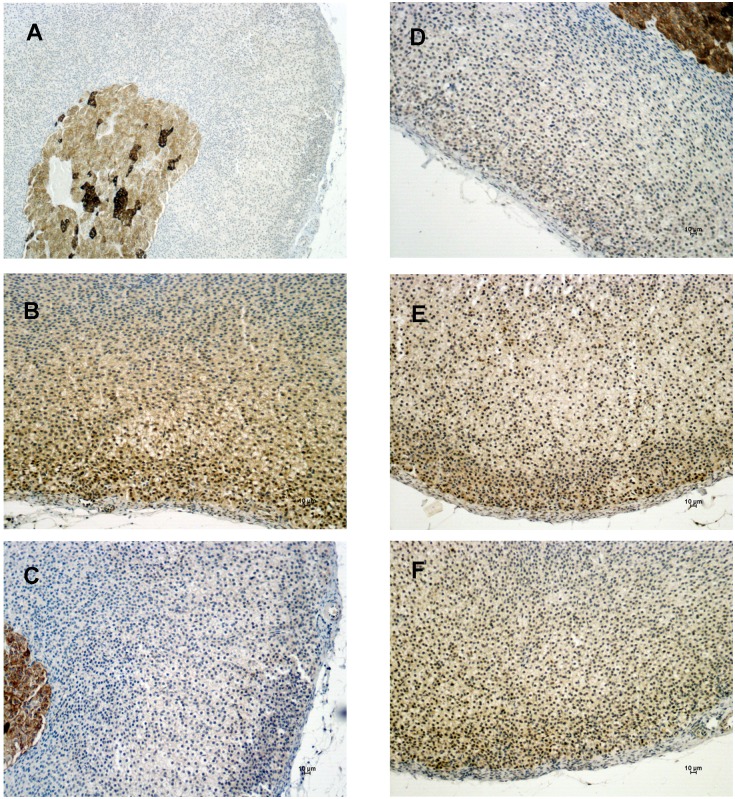
Immunolocalization of Notch3 expression in the adrenal cortex. Adrenal sections were obtained from rats treated with saline only (A and B) or with DEX for two days (50 µg/100 g BW) followed by: saline (C), or ACTH (D), or N-POMC^Met^ (E), or even N-POMC^Cys^ (F). Notch3-positive cells are stained in brown and indicated by arrows. Sections were counterstained with Harris’ hematoxylin and differentiated with a saturated solution of lithium carbonate. Bars 10 µm.

Our PCR array data showed that *Notch2* was up-regulated in the ZG and ZF fractions after ACTH or N-POMC^Met^, respectively. In order to determine the expression of the Notch2 protein, we performed an immunohistochemistry assay. Immunohistochemistry labeling for Notch2 showed no positive cells in the adrenal cortex or medulla, regardless of treatment. Positive Notch2 cells were present only in the adrenal capsule, and there was no modulation in their expression, irrespectively of treatment (Figure 6A). This result led us to evaluate the expression of two other Notch proteins in the adrenal gland, Notch1 and Notch3, even though we did not previously analyze their gene expression in the PCR array. The immunohistochemistry results for Notch1 revealed that this protein is expressed in both adrenal cortex and medulla (Figure 7A). Also, the expression of Notch1 is slightly reduced in the ZG of the cortex after HPA-axis inhibition with DEX (Figure 7B and C). ACTH and N-POMC^Met^ treatments promoted an increase in Notch1 expression in sub-capsular cells (Figure 7D and E), whereas N-POMC^Cys^ treatment did not alter its expression anywhere in the cortex (Figure 7F). Notch3 protein expression analyses revealed that the adrenal medulla has remarkable positive staining for this protein, when compared to the cortex (Figure 8A). Further, the inhibition of the HPA axis by DEX promoted a decrease in the levels of Notch3 protein expression in the entire cortex (Figure 8B and C). After ACTH treatment, there was no significant modification in the expression of this protein (Figure 8D). However, after N-POMC^Met^ or N-POMC^Cys^ treatments, we measured an increase in Notch3 expression (Figure 8E and F). The induction of Notch3 by N-POMC^Met^ or N-POMC^Cys^ did not reestablish the Notch3 expression observed in the control with saline only.

## Discussion

In this study we found that three genes (*Ccnb2*, *Camk2a*, and *Nek2*) were negatively regulated throughout the adrenal cortex when the HPA axis was inhibited. *Ccnb2* and *Camk2a* are related to the G2/M transition and *Nek2* is associated with key processes of spindle checkpoint. Cyclin B2/CDK1 is a key kinase complex involved in the transition of phase G2/M and its inhibition. Cyclins B1 and B2 act at the same CDK complex, promoting the progression of the cell cycle, and cyclin B2 is able to compensate for the down-regulation of cyclin B1 during mitosis in human cells [14]. Cyclin B2 has been shown to be overexpressed in adrenocortical carcinoma and is thus a good candidate for distinguishing benign from malignant adrenocortical tumors [15]. Calcium/Calmodulin-Dependent Protein Kinase II Alpha (Camk2a) is essential for regulating G2/M and the metaphase-anaphase transition in many cell types [16]. It is a member of the family of serine/threonine protein kinases and is a downstream effector of the angiotensin II-triggered signaling cascade that synthesizes and releases aldosterone in the ZG-cells [17]. Moreover, aldosterone production stimulated by ACTH in ZG cells is triggered by an increase in Ca^2+^ influx, which activates Camk signaling [18]. Therefore, the repression of the HPA axis could, at least in part, compromise the production of aldosterone in ZG by down-regulating the expression of *Camk2a*.

Equally important is *NEK* gene expression, which encodes a serine/threonine enzyme involved in key processes such as the centrosome cycle, kinetochore–microtubule attachment, spindle checkpoint/assembly, chromosome condensation, and cytokinesis (for a review, see [19]). Furthermore, overexpression of NEK2 has been associated with breast tumorigenesis [20]. In 2009, Giordano et al. published a microarray dataset for adrenocortical tumors (carcinomas and adenomas) and normal adrenals [21] (freely available on the NCBI website under Gene Expression Omnibus Series accession number GSE10927, www.ncbi.nlm.nih.gov/geo/). When we analyzed these data, we observed that *NEK2* is overexpressed in carcinomas compared to adenomas and normal adrenals (Figure S4). To our knowledge, this is the first report showing that Nek2 expression is associated with cell cycle regulation in adrenal glands.

Also, studies that screened the gene expression profile of serum-stimulated human fibroblasts showed that *Nek2* mRNA levels are highest in S and G2 [22]. In the current study, we showed that *Nek2b* gene and protein are induced in both adrenal fractions after treatments with ACTH or N-POMC^Met^, suggesting that *Nek2b* could be associated with the control of adrenocortical cell proliferation, since these treatments promoted an increase in the number of cells in the S-phase [7]. To the best of our knowledge, this is the first report showing that Nek2 expression is associated with cell cycle regulation in adrenal glands.

Following HPA-axis inhibition, there was an increase in the expression of Tumor protein p53 (*Tp53*) and Cyclin-dependent kinase 1A (*Cdkn1a*) in the ZG fraction. Both of these genes are associated with cell cycle arrest. In the ZG fraction, the absence of POMC-derived peptides does not promote a decrease in the number of cells in the S-phase, even with the increase of these two genes associated with cell cycle arrest. On the other hand, the *Wee1* gene was up-regulated in the ZF fraction. Wee1 kinase is able to phosphorylate and inhibit the cyclin B/CDK complex, which causes cell cycle arrest [23]. These data are in agreement with our own results showing the down-regulation of the cyclin B gene at the same time point and in the same adrenal fraction. Curiously, no gene was up-regulated in either fraction, reflecting the distinct peptide responses among adrenal cortex cells. The analysis of gene expression data plotted in the rat cell cycle pathway from the Kyoto Encyclopedia of Genes and Genomes (KEGG) shows that most cells in the ZG and ZF fractions seem to be in a different phase of the cell cycle, when analyzed at the same time point (Figures S2 and S3). Indeed, in the ZG fraction, HPA-axis inhibition down-regulates genes that are mainly related to the G1-S phases of the cell cycle, whereas in the ZF fraction, most repressed genes are in the G2 phase. Therefore, these data suggest that the zones comprising the adrenal cortex respond to inhibition of the HPA axis at different time points. This reinforces the notion of the independence of HPA axis control over the different zones of the adrenal cortex [24].

Regarding the effects of POMC-derived peptides in the adrenal cortex, we previously demonstrated that N-POMC^Met^ peptide and ACTH promoted an increase in the number of cells in S-phase in all adrenal zones, whereas N-POMC^Cys^ was effective only in ZG and ZR [7]. These data are in contrast with previous observations in POMC-null mice in which peripheral administration of N-POMC peptide did not change adrenal growth, but caused a reduction in food intake and body weight [25]. Moreover, results published previously by the same group [26] showed that the synthetic peptide N-POMC^Cys^ is able to stimulate cell proliferation of tumor adrenocortical cells and primary culture of bovine adrenal glands. In addition, results from our group, which used primary cultures of rat adrenal cells, detected the involvement of MAPK-ERK signaling after stimulation with N-POMC peptides [9]. Consistent with these results, we showed that only N-POMC^Met^ and ACTH up-regulated the expression of Nek2b in ZG and ZF, which highlights the differences between the two N-POMC peptides. Moreover, we observed that these mitotic treatments are able to increase the expression of genes related to the cohesin protein complex (Figure S3), which is associated with a late phase of cell division (for review see [27]). Interestingly, in N-POMC^Cys^ treatments, which do not stimulate ZF to enter the S-phase [7], the expression of the cohesin protein complex was not modulated.

The amino acid sequence of the N-terminal region of POMC is highly conserved between mammals [28], suggesting an important biological role for this peptide. The disulfide bridges in the N-terminal region of the native molecule induce the formation of a tertiary hairpin structure, which appears to be essential for accurate targeting of the peptide to the secretory region of the cell [29]. Here, the comparative analysis of the peptides’ effects on the expression of cell cycle genes in the ZG fraction, suggest that ZG cells are driven to different stages of the cell cycle, depending on the treatment (2h-ACTH or 2h-N-POMC). In fact, while 2h-ACTH treatment induced up-regulation of genes related to the G1-S transition, both 2h-N-POMC peptides down-regulated the genes associated with this phase. On the other hand, both 2h-N-POMC peptides up-regulated genes associated with the S-phase (*Pcna* and *Gadd45*; Figure S2).

The differences in gene expression profiles between the ZF and ZG fractions might be explained by the composition of the extracellular matrix (ECM) and the microenvironment of the adrenal zones [30]. There is some evidence that the ECM and integrins are differentially expressed throughout the rat adrenal cortex, and that the proliferative effect *in* *vitro* of ACTH in ZG and ZF cells depends on the composition of the substrate that these cells are exposed to [30]. Also, the vascularization of the adrenal gland, which begins at the periphery of the organ and reaches the marrow centripetally, promotes a diffusion gradient between the adrenal zones [31]. This diffusion gradient might be directly reflected in the concentration of POMC-derived peptides that each zone is influenced by, resulting in the observed differences in gene expression profiles.

Regarding Notch protein expression, we observed higher Notch1 expression in the subcapsular cells than in the rest of the cortex after ACTH and N-POMC^Met^ treatments, but not after N-POMC^Cys^ treatment. Moreover, Notch2 appears to be expressed only in the capsular cells, irrespective of the treatment. The subcapsular and capsular cells of the adrenal cortex have been considered to be the main site of undifferentiated cells, involved in adrenocortical zonation, remodeling, and homeostasis (for a review, see [32]). It has recently been shown that Delta-like homologue 1 (Dlk1), which physically interacts with Notch1 [33], is expressed in the subcapsular region of the cortex and stimulates Gli1 expression in the mesenchymal cells of the adrenal capsule [34]. Therefore, after proliferative treatments, Notch1 is expressed in the same subcapsular region as Dlk1 and Sonic-Hedgehog (Shh), which suggests that this protein might be involved in the regulation of adrenocortical remodeling and homeostasis. Further studies must be conducted to elucidate the role of Notch1 in subcapsular cells. The Notch signaling pathway is one of the most altered pathways regarding adrenal tumors, suggesting that this pathway is also crucial for the proliferation of adrenocortical cells [35]. Interestingly, Notch3 is highly expressed in the adrenal medulla, where the presence of chromaffin progenitor cells has already been reported [36].

In conclusion, our study shows that the inhibition of the HPA axis represses the expression of *Ccnb2*, *Camk2a*, and *Nek2* genes throughout the adrenal cortex, and that *Nek2* is up-regulated after treatments with proliferative POMC-derived peptides. We also demonstrated that the zones of the adrenal cortex respond to HPA-axis inhibition and POMC-derived peptides at different times, reinforcing the notion that cells from the ZG and ZF fractions are in different phases of the cell cycle. Moreover, our results contribute to the understanding of the mechanisms triggered by N-POMC peptides and ACTH in genes associated with cell cycle regulation in adrenocortical cells.

## Supporting Information

Figure S1 **Isoforms of Nek2 protein in commercial HeLa nuclear extracts, Y1 total extracts, rat adrenal gland extracts, and primary rat adrenal cell culture.** Total proteins from Y1 cells, rat adrenal gland, and primary rat adrenal cell culture were extracted by using RIPA buffer.
(TIF)

Figure S2 **Analysis of gene expression data from the ZG fraction plotted in rat cell cycle pathway from the Kyoto Encyclopedia of Genes and Genomes (KEGG).** The genes highlighted in green are down-regulated and those in red are up-regulated.
(TIF)

Figure S3 **Analysis of gene expression data from the ZF fraction plotted in rat cell cycle pathway from the Kyoto Encyclopedia of Genes and Genomes (KEGG).** The genes highlighted in green are down-regulated and those in red are up-regulated.
(TIF)

Figure S4 **The expression of** ***NEK2*** **comparing the microarray dataset from adrenocortical tumors, carcinomas and adenomas, and normal adrenals.** The microarray dataset is published in [21] and is freely available on the website www.ncbi.nlm.nih.gov/geo/through Gene Expression Omnibus Series accession number GSE10927.
(TIF)

Table S1 **List of all genes analyzed in the RT-PCR-array (Rat Cell Cycle PARN-020 from Sabiosciences – Qiagen).**
(DOC)
