# Supplementary figures and images for: The Involvement of Nek2 and Notch in the Proliferation of Rat Adrenal Cortex Triggered by POMC-Derived Peptides
Source: PLoS One. 2014 Oct 3;9(10):e108657. doi: 10.1371/journal.pone.0108657 (PMC4184836; doi:10.1371/journal.pone.0108657)

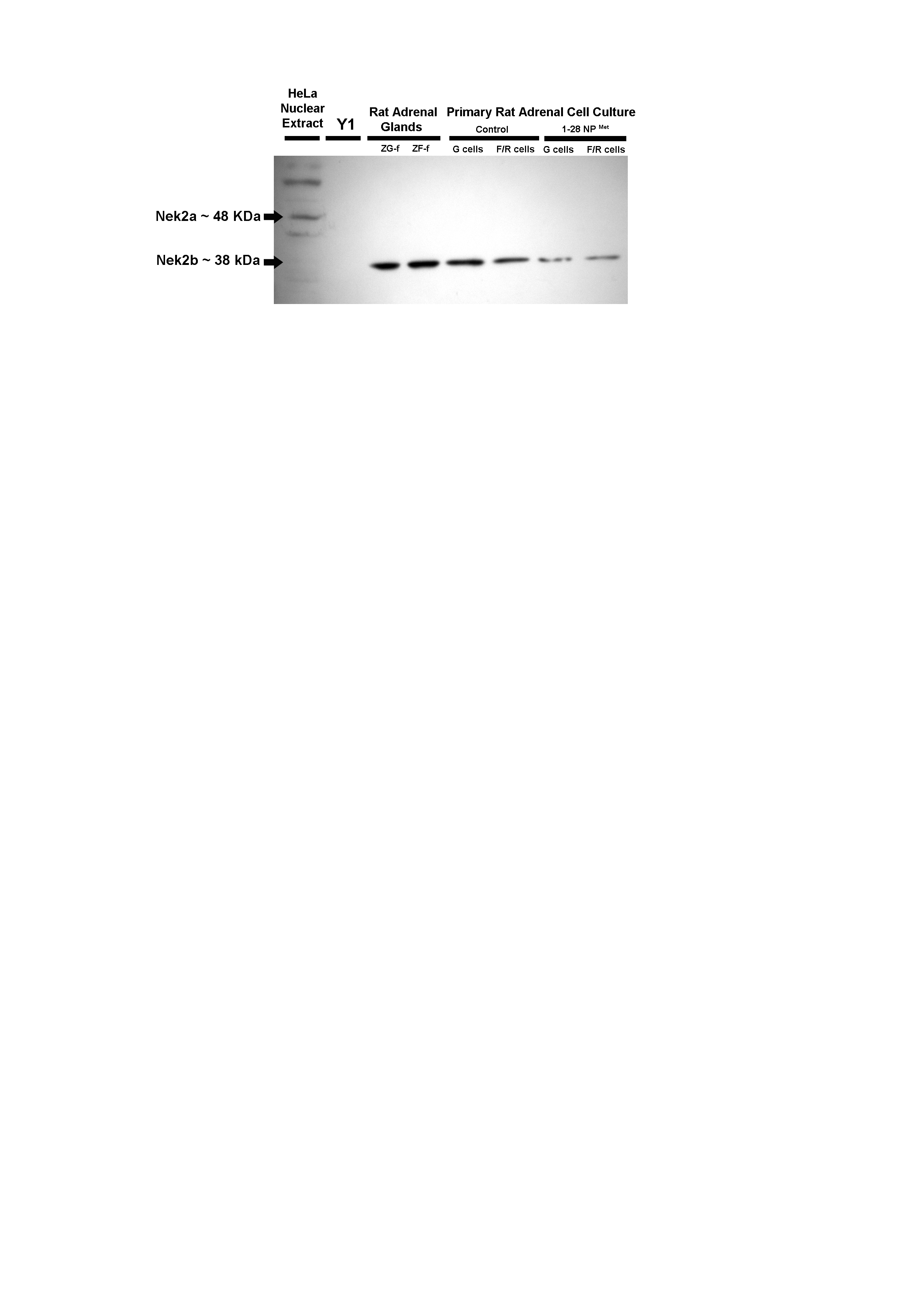

Supplement: Figure S1 — Isoforms of Nek2 protein in commercial HeLa nuclear extracts, Y1 total extracts, rat adrenal gland extracts, and primary rat adrenal cell culture. Total proteins from Y1 cells, rat adrenal gland, and primary rat adrenal cell culture were extracted by using RIPA buffer. (TIF) [file pone.0108657.s001.tif]

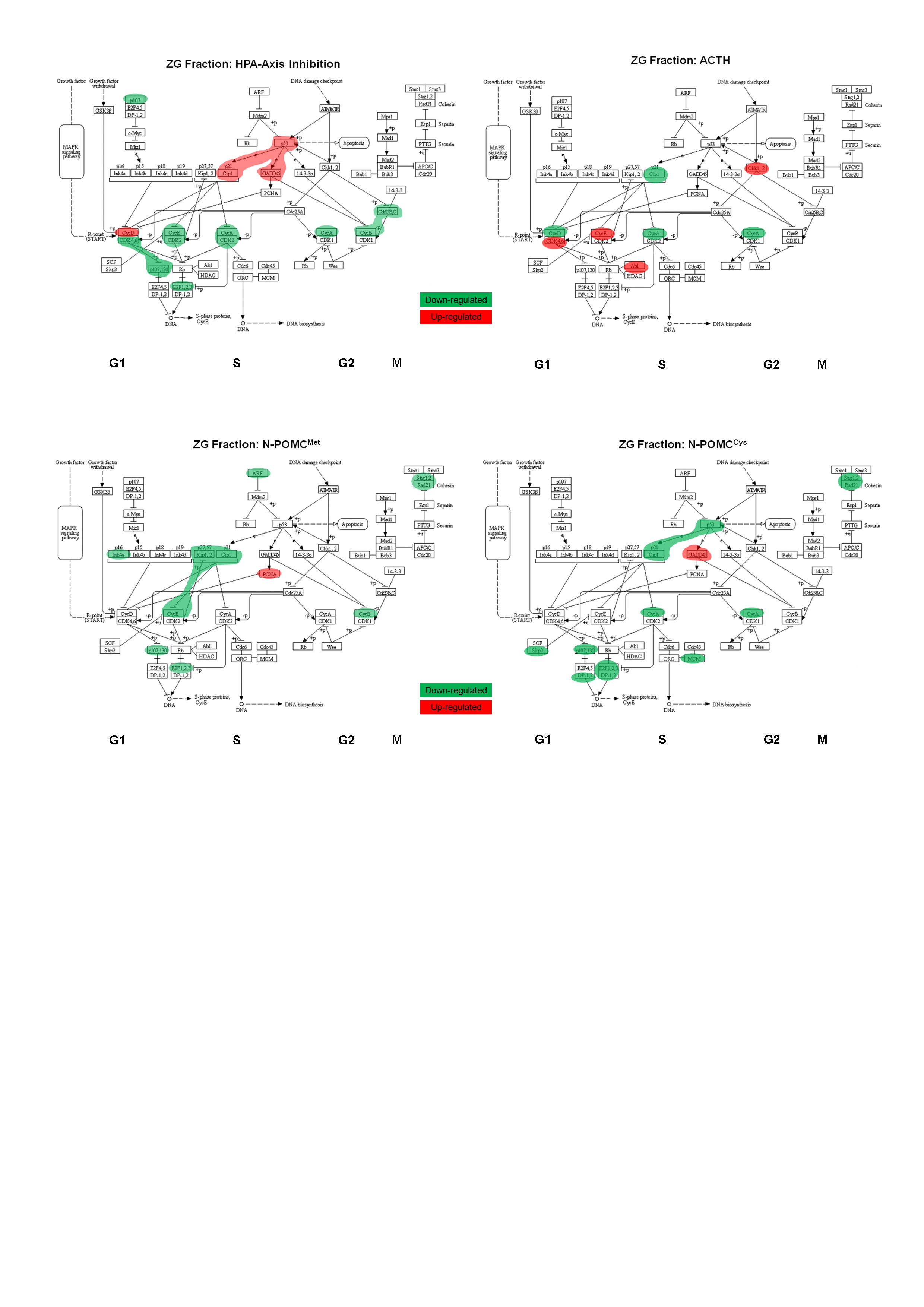

Supplement: Figure S2 — Analysis of gene expression data from the ZG fraction plotted in rat cell cycle pathway from the Kyoto Encyclopedia of Genes and Genomes (KEGG). The genes highlighted in green are down-regulated and those in red are up-regulated. (TIF) [file pone.0108657.s002.tif]

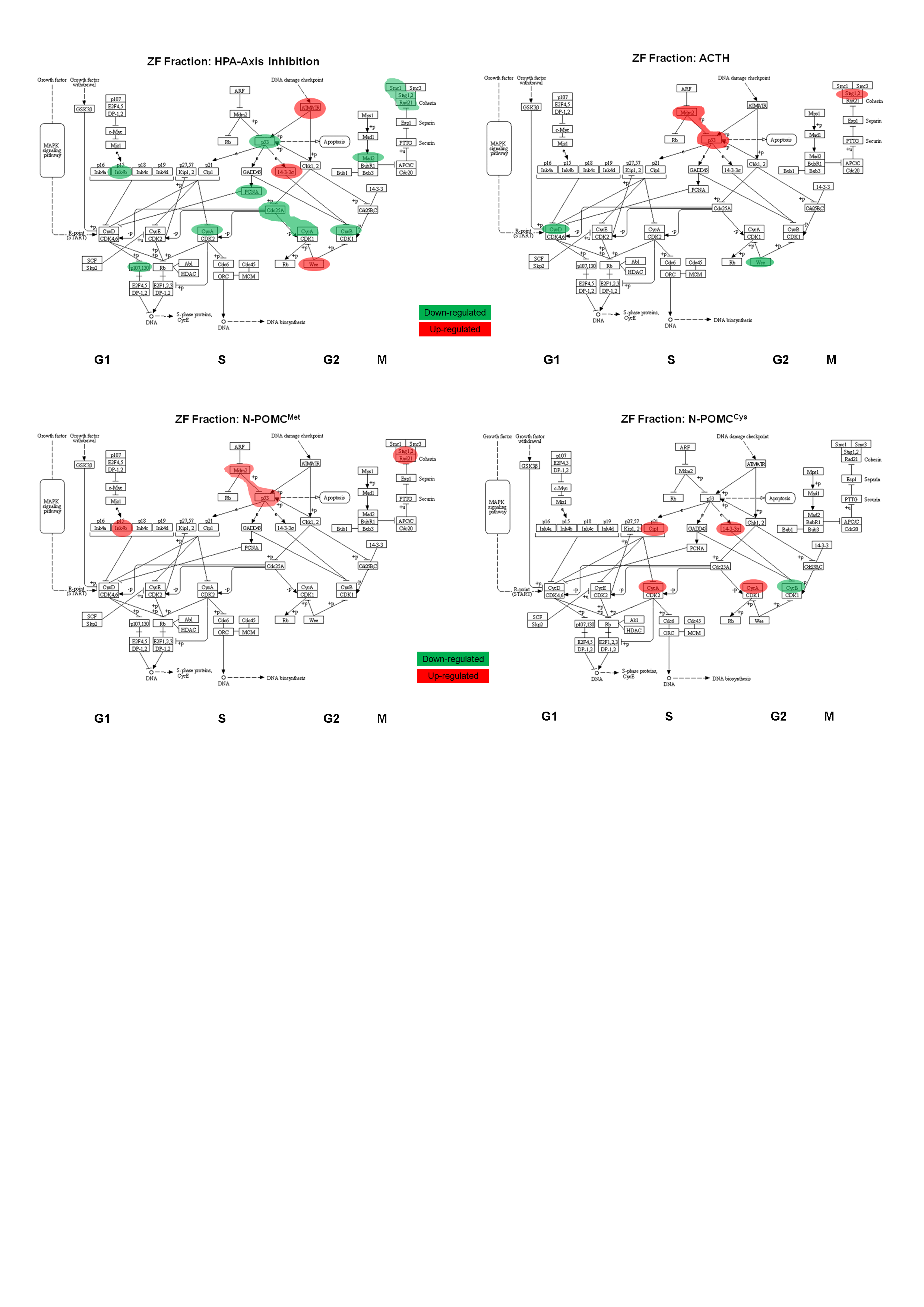

Supplement: Figure S3 — Analysis of gene expression data from the ZF fraction plotted in rat cell cycle pathway from the Kyoto Encyclopedia of Genes and Genomes (KEGG). The genes highlighted in green are down-regulated and those in red are up-regulated. (TIF) [file pone.0108657.s003.tif]

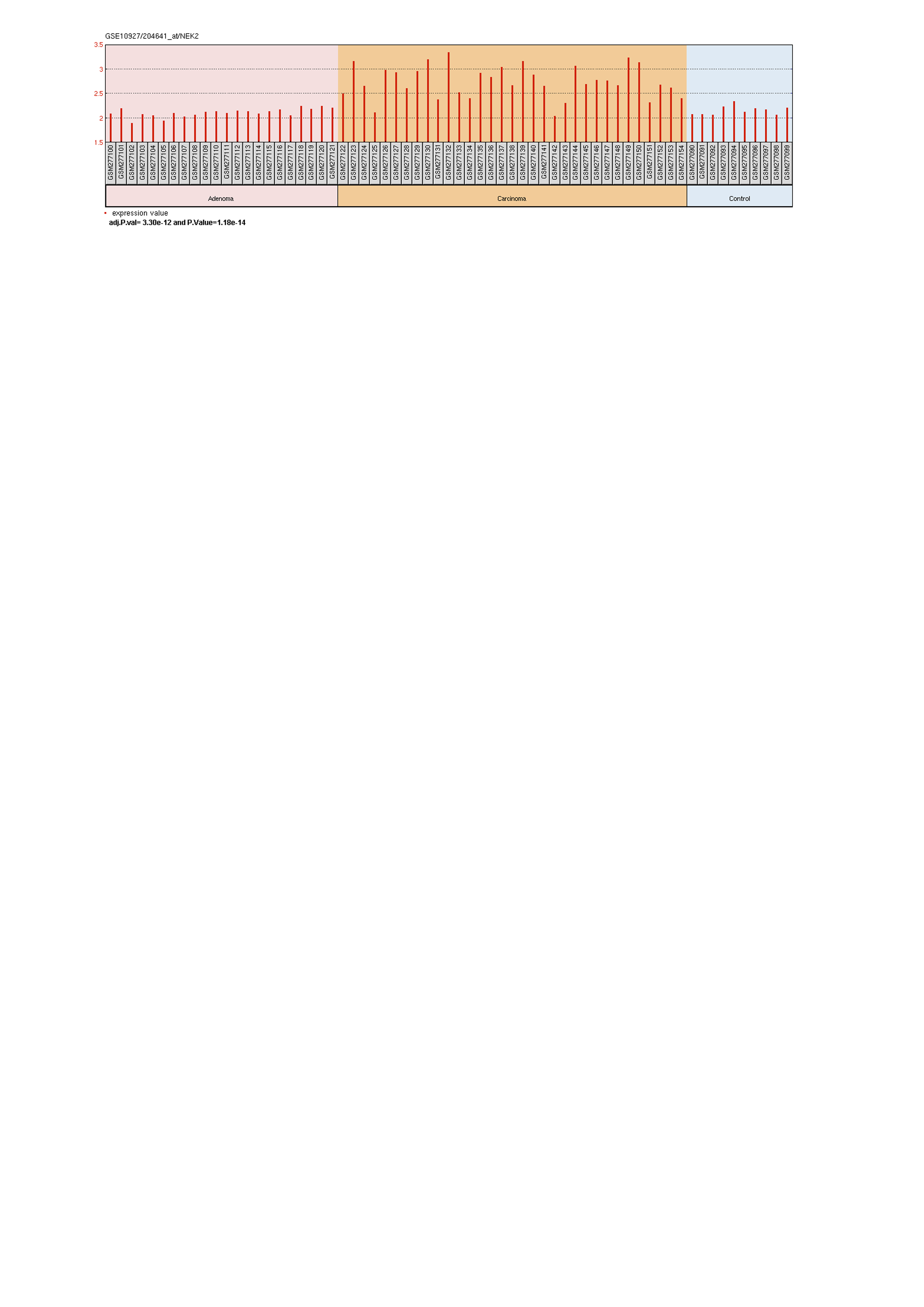

Supplement: Figure S4 — The expression of NEK2 comparing the microarray dataset from adrenocortical tumors, carcinomas and adenomas, and normal adrenals. The microarray dataset is published in [21] and is freely available on the website www.ncbi.nlm.nih.gov/geo/through Gene Expression Omnibus Series accession number GSE10927. (TIF) [file pone.0108657.s004.tif]
