# Supplementary material for: The Involvement of Nek2 and Notch in the Proliferation of Rat Adrenal Cortex Triggered by POMC-Derived Peptides
Source: PLoS One. 2014 Oct 3;9(10):e108657. doi: 10.1371/journal.pone.0108657 (PMC4184836; doi:10.1371/journal.pone.0108657)
Supplement: Table S1 — List of all genes analyzed in the RT-PCR-array (Rat Cell Cycle PARN-020 from Sabiosciences – Qiagen). (DOC) [file pone.0108657.s005.doc]

Table S1. List of all genes analyzed in the RT-PCR-array.

| **Description** | **Symbol** | **Gene Name** | **Unigene** | **GeneBank** |
| --- | --- | --- | --- | --- |
| **C-abl oncogene 1, receptor tyrosine kinase** | ***Abl1*** | **Abl** | **Rn.3105** | **NM_001100850** |
| **Adenylate kinase 1** | ***Ak1*** | **MGC108678** | **Rn.79537** | **NM_024349** |
| **Amyloid beta (A4) precursor protein-binding, family B, member 1 (Fe65)** | ***Apbb1*** | **FE65** | **Rn.19953** | **NM_080478** |
| **Ataxia telangiectasia mutated homolog (human)** | ***Atm*** | **-** | **Rn.214048** | **NM_001106821** |
| **Breast cancer 1** | ***Brca1*** | **-** | **Rn.48840** | **NM_012514** |
| **Breast cancer 2** | ***Brca2*** | **-** | **Rn.103225** | **NM_031542** |
| **Calcium/calmodulin-dependent protein kinase II alpha** | ***Camk2a*** | **PK2CDD, PKCCD** | **Rn.107499** | **NM_012920** |
| **Calcium/calmodulin-dependent protein kinase II beta** | ***Camk2b*** | **Ck2b** | **Rn.9743** | **NM_021739** |
| **Caspase 3** | ***Casp3*** | **Lice, MGC93645** | **Rn.10562** | **NM_012922** |
| **Cyclin A1** | ***Ccna1*** | **-** | **Rn.102823** | **NM_001011949** |
| **Cyclin A2** | ***Ccna2*** | **MGC156527** | **Rn.13094** | **NM_053702** |
| **Cyclin B1** | ***Ccnb1*** | **-** | **Rn.9232** | **NM_171991** |
| **Cyclin B2** | ***Ccnb2*** | **MGC108931** | **Rn.6743** | **NM_001009470** |
| **Cyclin C** | ***Ccnc*** | **-** | **Rn.106758** | **XM_342812** |
| **Cyclin D1** | ***Ccnd1*** | **-** | **Rn.22279** | **NM_171992** |
| **Cyclin D2** | ***Ccnd2*** | **-** | **Rn.96083** | **NM_022267** |
| **Cyclin E1** | ***Ccne1*** | **CYCLE, Ccne** | **Rn.15455** | **NM_001100821** |
| **Cyclin F** | ***Ccnf*** | **-** | **Rn.15126** | **NM_001100474** |
| **Cell division cycle 25 homolog A (*S. pombe*)** | ***Cdc25a*** | **-** | **Rn.11390** | **NM_133571** |
| **Cell division cycle 25 homolog B (*S. pombe*)** | ***Cdc25b*** | **-** | **Rn.11312** | **NM_133572** |
| **Cyclin dependent kinase 2** | ***Cdk2*** | **-** | **Rn.104460** | **NM_199501** |
| **Cyclin-dependent kinase 4** | ***Cdk4*** | **-** | **Rn.6115** | **NM_053593** |
| **CDK5 regulatory subunit associated protein 1** | ***Cdk5rap1*** | **-** | **Rn.203271** | **NM_145721** |
| **Cyclin-dependent kinase inhibitor 1A** | ***Cdkn1a*** | **Cip1, Waf1** | **Rn.10089** | **NM_080782** |
| **Cyclin-dependent kinase inhibitor 1B** | ***Cdkn1b*** | **CDKN4, Cdki1b, Kip1, P27KIP1, p27** | **Rn.29897** | **NM_031762** |
| **Cyclin-dependent kinase inhibitor 2A** | ***Cdkn2a*** | **Arf, INK4A, MTS1, p16, p16Cdkn2a, p19ARF** | **Rn.48717** | **NM_031550** |
| **Cyclin-dependent kinase inhibitor 2B (p15, inhibits CDK4)** | ***Cdkn2b*** | **p15** | **Rn.105626** | **NM_130812** |
| **CHK1 checkpoint homolog (*S. pombe*)** | ***Chek1*** | **-** | **Rn.33267** | **NM_080400** |
| **DNA-damage inducible transcript 3** | ***Ddit3*** | **CHOP, CHOP-10, Chop10, Gadd153, MGC124604** | **Rn.11183** | **NM_024134** |
| **DnaJ (Hsp40) homolog, subfamily C, member 2** | ***Dnajc2*** | **MGC105894, MIDA1, Zrf2** | **Rn.11908** | **NM_053776** |
| **Dystonin** | ***Dst*** | **-** | **Rn.79807** | **NM_001108208** |
| **E2F transcription factor 1** | ***E2f1*** | **-** | **Rn.72471** | **NM_001100778** |
| **E2F transcription factor 4** | ***E2f4*** | **-** | **Rn.154586** | **XM_226441** |
| **Growth arrest and DNA-damage-inducible, alpha** | ***Gadd45a*** | **Ddit1, Gadd45** | **Rn.10250** | **NM_024127** |
| **G protein-coupled receptor 132** | ***Gpr132*** | **G2a** | **Rn.145095** | **XM_234574** |
| **Inhibin alpha** | ***Inha*** | **MGC93593** | **Rn.8831** | **NM_012590** |
| **Integrin, beta 1** | ***Itgb1*** | **-** | **Rn.25733** | **NM_017022** |
| **Pescadillo homolog 1, containing BRCT domain (zebrafish)** | ***Pes1*** | **MGC125075** | **Rn.9521** | **NM_001044228** |
| **E2F transcription factor 3** | ***E2f3*** | **RGD1561600** | **Rn.73967** | **XM_214476** |
| **Similar to nuclear factor of activated T-cells, cytoplasmic, calcineurin-dependent 1** | ***LOC307231*** | **-** | **Rn.8800** | **XM_225713** |
| **Nanos homolog 2 (Drosophila)** | ***Nanos2*** | **RGD1562436** | **Rn.218571** | **NM_001108908** |
| **Similar to DNA replication licensing factor MCM3 (DNA polymerase alpha holoenzyme-associated protein P1) (P1-MCM3)** | ***LOC367976*** | **-** | **Rn.154788** | **XM_346381** |
| **HUS1 checkpoint homolog (*S. pombe*)** | ***Hus1*** | **-** | **Rn.12812** | **NM_001109092** |
| **B-cell CLL/lymphoma 2** | ***Bcl2*** | **Bcl-2** | **Rn.9996** | **NM_016993** |
| **Schlafen 1** | ***Slfn1*** | **RGD1309755** | **Rn.211782** | **XM_001068751** |
| **MAD2 mitotic arrest deficient-like 1 (yeast)** | ***Mad2l1*** | **-** | **Rn.19771** | **XM_216161** |
| **Minichromosome maintenance complex component 2** | ***Mcm2*** | **-** | **Rn.2715** | **NM_001107873** |
| **Minichromosome maintenance complex component 4** | ***Mcm4*** | **Mcmd4** | **Rn.8341** | **NM_033651** |
| **Mdm2 p53 binding protein homolog (mouse)** | ***Mdm2*** | **-** | **Rn.91829** | **NM_001108099** |
| **RAD21 homolog (*S. pombe*)** | ***Rad21*** | **MGC116373** | **Rn.3991** | **NM_001025701** |
| **Antigen identified by monoclonal antibody Ki-67** | ***Mki67*** | **-** | **Rn.73551** | **XM_225460** |
| **MRE11 meiotic recombination 11 homolog A (*S. cerevisiae*)** | ***Mre11a*** | **Mre11** | **Rn.209040** | **NM_022279** |
| **MutS homolog 2 (*E. coli*)** | ***Msh2*** | **-** | **Rn.3174** | **NM_031058** |
| **NIMA (never in mitosis gene a)-related expressed kinase 2** | ***Nek2*** | **-** | **Rn.144627** | **NM_053691** |
| **Notch homolog 2 (Drosophila)** | ***Notch2*** | **-** | **Rn.65930** | **NM_024358** |
| **Nucleophosmin/nucleoplasmin 2** | ***Npm2*** | **-** | **Rn.214645** | **NM_203340** |
| **Proliferating cell nuclear antigen** | ***Pcna*** | **PCNAR, Pcna, cyclin** | **Rn.223** | **NM_022381** |
| **Polycystic kidney disease 1 homolog (human)** | ***Pkd1*** | **-** | **Rn.127770** | **XM_002724589** |
| **Peripheral myelin protein 22** | ***Pmp22*** | **Gas-3** | **Rn.1476** | **NM_017037** |
| **Protein phosphatase 1D magnesium-dependent, delta isoform** | ***Ppm1d*** | **-** | **Rn.15540** | **NM_001105825** |
| **Protein phosphatase 2, regulatory subunit B'', alpha** | ***Ppp2r3a*** | **-** | **Rn.93024** | **NM_001012202** |
| **Protein phosphatase 3, catalytic subunit, alpha isoform** | ***Ppp3ca*** | **Calna1** | **Rn.6866** | **NM_017041** |
| **Protamine 1** | ***Prm1*** | **-** | **Rn.27657** | **NM_001002850** |
| **RAD17 homolog (*S. pombe*)** | ***Rad17*** | **-** | **Rn.154275** | **NM_001024778** |
| **RAD51 homolog (RecA homolog, *E. coli*) (*S. cerevisiae*)** | ***Rad51*** | **RGD1563603** | **Rn.214052** | **NM_001109204** |
| **RAD9 homolog (*S. pombe*)** | ***Rad9*** | **-** | **Rn.145016** | **XM_219684** |
| **RAN, member RAS oncogene family** | ***Ran*** | **-** | **Rn.107698** | **NM_053439** |
| **Retinoblastoma-like 1 (p107)** | ***Rbl1*** | **-** | **Rn.208977** | **XM_001055763** |
| **Retinoblastoma-like 2** | ***Rbl2*** | **Rb2** | **Rn.11020** | **NM_031094** |
| **S-phase kinase-associated protein 2 (p45)** | ***Skp2*** | **RGD1562456** | **Rn.154278** | **NM_001106416** |
| **Similar to Sestrin 2 (Hi95)** | ***RGD1566319*** | **-** | **Rn.216753** | **NM_001109358** |
| **Stratifin** | ***Sfn*** | **-** | **Rn.145079** | **XM_232745** |
| **SHC (Src homology 2 domain containing) transforming protein 1** | ***Shc1*** | **P66shc** | **Rn.138818** | **NM_053517** |
| **Structural maintenance of chromosomes 1A** | ***Smc1a*** | **KIAA0178, SB1.8, SMC-1A, Smc1l1** | **Rn.11763** | **NM_031683** |
| **Stromal antigen 1** | ***Stag1*** | **-** | **Rn.11715** | **NM_001108179** |
| **SMT3 suppressor of mif two 3 homolog 1 (S. cerevisiae)** | ***Sumo1*** | **MGC109561** | **Rn.1221** | **NM_001009672** |
| **TAF10 RNA polymerase II, TATA box binding protein (TBP)-associated factor** | ***Taf10*** | **MGC188059** | **Rn.144795** | **NM_001134735** |
| **Telomeric repeat binding factor (NIMA-interacting) 1** | ***Terf1*** | **MGC109063** | **Rn.33853** | **NM_001012464** |
| **Transcription factor Dp-2 (E2F dimerization partner 2)** | ***Tfdp2*** | **Tcfdp2** | **Rn.105731** | **NM_001106847** |
| **Proteasome (prosome, macropain) assembly chaperone 2** | ***Psmg2*** | **Tnfsf5ip1** | **Rn.144759** | **NM_001106138** |
| **Tumor protein p53** | ***Tp53*** | **MGC112612, Trp53, p53** | **Rn.54443** | **NM_030989** |
| **Tumor protein p63** | ***Tp63*** | **Ket, P73l, Tp73l, Trp63** | **Rn.42907** | **NM_019221** |
| **Tumor susceptibility gene 101** | ***Tsg101*** | **Rw** | **Rn.7410** | **NM_181628** |
| **Wee 1 homolog (*S. pombe*)** | ***Wee1*** | **MGC105683** | **Rn.208255** | **NM_001012742** |
| **Ribosomal protein, large, P1** | ***Rplp1*** | **MGC72935** | **Rn.973** | **NM_001007604** |
| **Hypoxanthine phosphoribosyltransferase 1** | ***Hprt1*** | **Hgprtase, Hprt, MGC112554** | **Rn.47** | **NM_012583** |
| **Ribosomal protein L13A** | ***Rpl13a*** | **-** | **Rn.92211** | **NM_173340** |
| **Lactate dehydrogenase A** | ***Ldha*** | **Ldh1** | **Rn.107896** | **NM_017025** |
| **Actin, beta** | ***Actb*** | **Actx** | **Rn.94978** | **NM_031144** |
| **Rat Genomic DNA Contamination** | ***RGDC*** | **RGDC** | **N/A** | **U26919** |
| **Reverse Transcription Control** | ***RTC*** | **RTC** | **N/A** | **SA_00104** |
| **Reverse Transcription Control** | ***RTC*** | **RTC** | **N/A** | **SA_00104** |
| **Reverse Transcription Control** | ***RTC*** | **RTC** | **N/A** | **SA_00104** |
| **Positive PCR Control** | ***PPC*** | **PPC** | **N/A** | **SA_00103** |
| **Positive PCR Control** | ***PPC*** | **PPC** | **N/A** | **SA_00103** |
| **Positive PCR Control** | ***PPC*** | **PPC** | **N/A** | **SA_00103** |
